# Enhanced in situ H_2_O_2_ production explains synergy between an LPMO with a cellulose-binding domain and a single-domain LPMO

**DOI:** 10.1038/s41598-022-10096-0

**Published:** 2022-04-12

**Authors:** Anton A. Stepnov, Vincent G. H. Eijsink, Zarah Forsberg

**Affiliations:** Faculty of Chemistry, Biotechnology and Food Science, NMBU - Norwegian University of Life Sciences, 1432 Ås, Norway

**Keywords:** Enzymes, Polysaccharides

## Abstract

Lytic polysaccharide monooxygenases (LPMOs) are mono-copper enzymes that catalyze oxidative depolymerization of recalcitrant substrates such as chitin or cellulose. Recent work has shown that LPMOs catalyze fast peroxygenase reactions and that, under commonly used reaction set-ups, access to in situ generated H_2_O_2_ likely limits catalysis. Based on a hypothesis that the impact of a cellulose-binding module (CBM) on LPMO activity could relate to changes in in situ H_2_O_2_ production, we have assessed the interplay between CBM-containing *Sc*LPMO10C and its truncated form comprising the catalytic domain only (*Sc*LPMO10C_TR_). The results show that truncation of the linker and CBM leads to elevated H_2_O_2_ production and decreased enzyme stability. Most interestingly, combining the two enzyme forms yields strong synergistic effects, which are due to the combination of high H_2_O_2_ generation by *Sc*LPMO10C_TR_ and efficient productive use of H_2_O_2_ by the full-length enzyme. Thus, cellulose degradation becomes faster, while enzyme inactivation due to off-pathway reactions with excess H_2_O_2_ is reduced. These results underpin the complexity of ascorbic acid-driven LPMO reactions and reveal a potential mechanism for how LPMOs may interact synergistically during cellulose degradation.

## Introduction

Lytic polysaccharide monooxygenases (LPMOs) are monocopper enzymes that catalyze oxidative depolymerization of polysaccharide substrates, such as chitin^1^, cellulose^2–4^ and xylan^5–7^. LPMOs depend on an electron source to maintain their catalytic cycle and possess a unique ability to act on crystalline surfaces of recalcitrant polysaccharides, making their targets prone to further degradation by classical hydrolytic enzymes^8^. The synergy between LPMOs, chitinases and cellulases has attracted significant attention not only in academia but also in industry^1,9–14^. Modern commercial cellulolytic cocktails, such as Cellic CTec2 and CTec3, benefit from the inclusion of LPMOs in their formulations^10,15^. 

LPMOs were previously thought to use molecular oxygen as a co-substrate, but it has recently been shown that these enzymes prefer hydrogen peroxide, thus operating as peroxygenases rather than strict monooxygenases^16^. Importantly, due to the preference for H_2_O_2_, the rates of LPMO reactions that are carried out in the absence of exogenously added hydrogen peroxide are limited by in situ H_2_O_2_ generation^16–18^ and are low compared to reactions with controlled addition of H_2_O_2_^17,19,20^.

Both non-enzymatic and enzyme-dependent pathways lead to hydrogen peroxide generation in typical LPMO reactions. Firstly, commonly used low-molecular-weight LPMO reductants, such as ascorbic acid, gallic acid and cysteine, are known to engage into direct reactions with dissolved molecular oxygen, yielding H_2_O_2_^21–23^. Secondly, it is well established that reduced LPMOs have oxidase activity, which leads to hydrogen peroxide production^24^ with rates that depend on the nature of the reductant that is being oxidized^17,18,25^. It is worth noting that, besides driving LPMO activity on carbohydrates, H_2_O_2_ may promote auto-catalytic damage to the active site of the LPMO, especially when substrate concentrations are low^16,26^. All in all, a detailed understanding of the factors determining the rate of in situ H_2_O_2_ generation in LPMO reactions is essential for unlocking the full potential of these enzymes.

One important but rather unexplored aspect of LPMO chemistry is the interplay between enzyme-dependent H_2_O_2_ production and substrate binding. A few recent studies involving fungal (family AA9) and bacterial (family AA10) LPMOs have confirmed early suggestions by Kittl et al.^24^ by showing that hydrogen peroxide generation by these enzymes is repressed in the presence of insoluble substrate^27,28^. Such observations make sense considering the fact that binding to a crystalline surface will shield the active site from the solvent to a degree that will prevent the interaction of low-molecular reductants with the copper atom^29^. In Nature, binding of LPMOs to insoluble polysaccharides is often guided by carbohydrate-binding modules (CBMs)^30^. Compared to LPMO catalytic domains, CBMs are capable of stronger binding to substrates^31,32^, and it has been shown that CBMs have pronounced impact on LPMO activity and stability because they contribute to keeping the active site closer to the substrate^32–34^.

Considering the above, it is reasonable to assume that the presence or absence of a CBM will affect hydrogen peroxide production by LPMOs in reactions with insoluble polysaccharide substrates, with potential repercussions on (H_2_O_2_-driven) LPMO activity. To study these issues, we have used a CBM-containing family AA10 LPMO from *Streptomyces coelicolor* (*Sc*LPMO10C) and its truncated form lacking the CBM and the linker (*Sc*LPMO10C_TR_) as model bacterial enzymes and studied the interplay and possible synergy between these. We show that the two enzyme forms act synergistically during cellulose degradation and, by studying H_2_O_2_ production and reductant depletion in various reaction set-ups, we show that this synergy relates to redox processes in the reaction mixture that affect co-substrate availability and oxidative enzyme inactivation. Next to shedding light on the possible interplay between CBM-containing and CBM-free LPMOs, our results provide fundamental insights into the complexity of LPMO reactions that are fueled by ascorbic acid, the most commonly used reductant in LPMO research.

## Results and discussion

### Protein production

**Figure 1:**
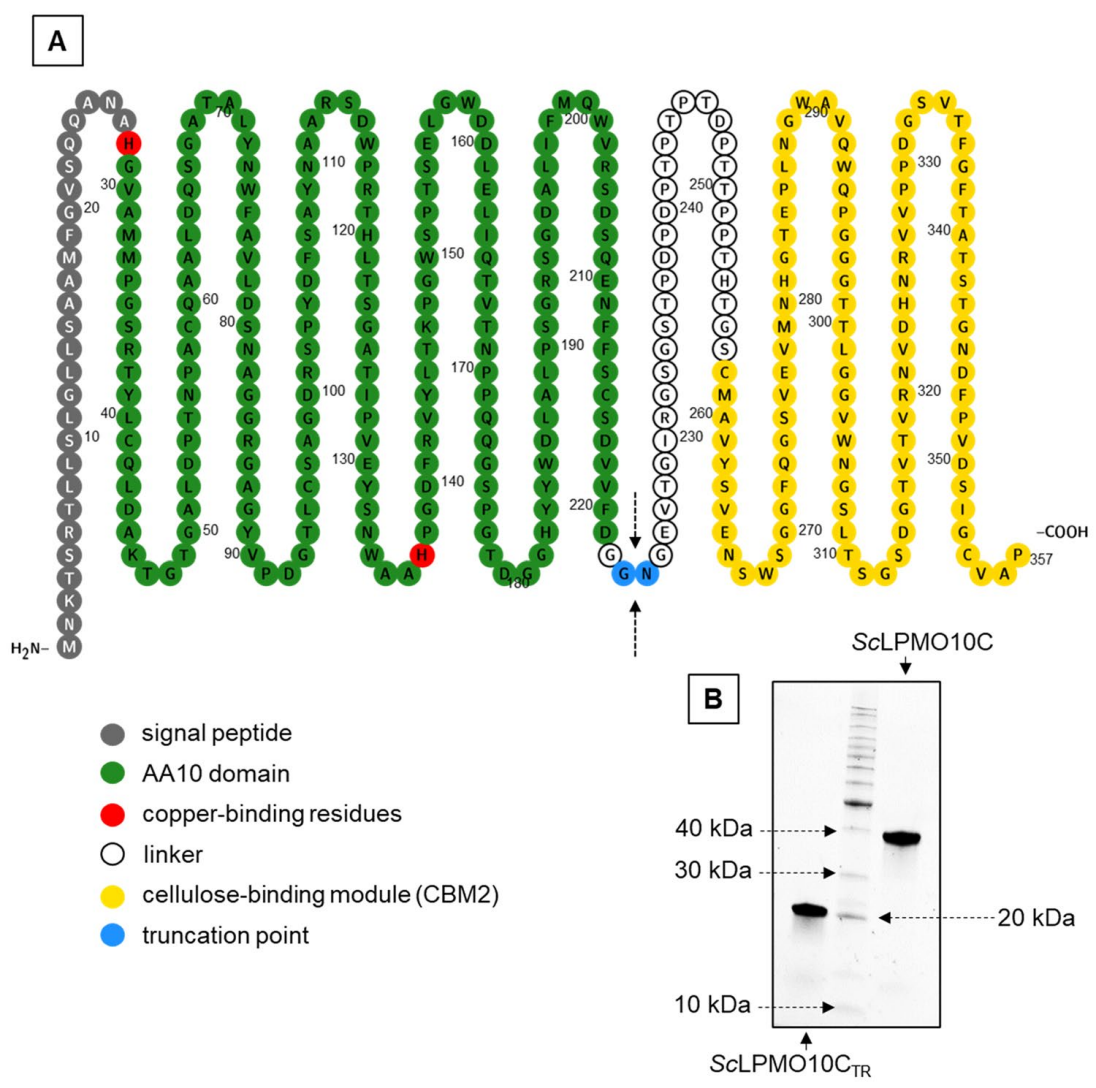
Domain architecture of *Sc*LPMO10C (panel **A**) and SDS-PAGE of purified *Sc*LPMO10C and *Sc*LPMO10C_TR_ (panel **B**). *Sc*LPMO10C_TR_ is a modified form of *Sc*LPMO10C produced by truncation of the cellulose-binding module as indicated by the blue color and the arrows. For expression, the native signal peptide was replaced by the signal peptide of *Sm*LPMO10A (CBP21), which is shown in the figure. Domain boundaries were annotated using Pfam (http://pfam.xfam.org/), with minor manual adjustments based on inspection of the crystal structure of the catalytic domain (PDB: 4OY7) and the NMR structure of the cellulose-binding domain (PDB: 6F7E). The schematic representation of *Sc*LPMO10C was created with the Protter protein visualization tool (http://wlab.ethz.ch/protter). See. Fig. S1 for the uncropped and unprocessed gel image used to create panel B.

To investigate the impact of the cellulose-binding module (CBM) on LPMO performance in reactions with insoluble substrate, a well-characterized enzyme from the Gram-positive soil bacterium *Streptomyces coelicolor*, *Sc*LPMO10C, was selected as the model. The LPMO and its truncated form lacking the linker and the CBM2, *Sc*LPMO10C_TR_ (Fig. 1A), were produced using previously described *E. coli* expression strains^35^. Both enzymes were isolated from the periplasm in soluble form and purified by ion-exchange and size-exclusion chromatography (Fig. 1B). The final yield amounted to approximately 60 mg of *Sc*LPMO10C and 20 mg of *Sc*LPMO10C_TR_ per liter of culture.

### Hydrogen peroxide generation by *Sc*LPMO10C and *Sc*LPMO10C_TR_ and assessment of free copper levels in the enzyme preparations

**Figure 2:**
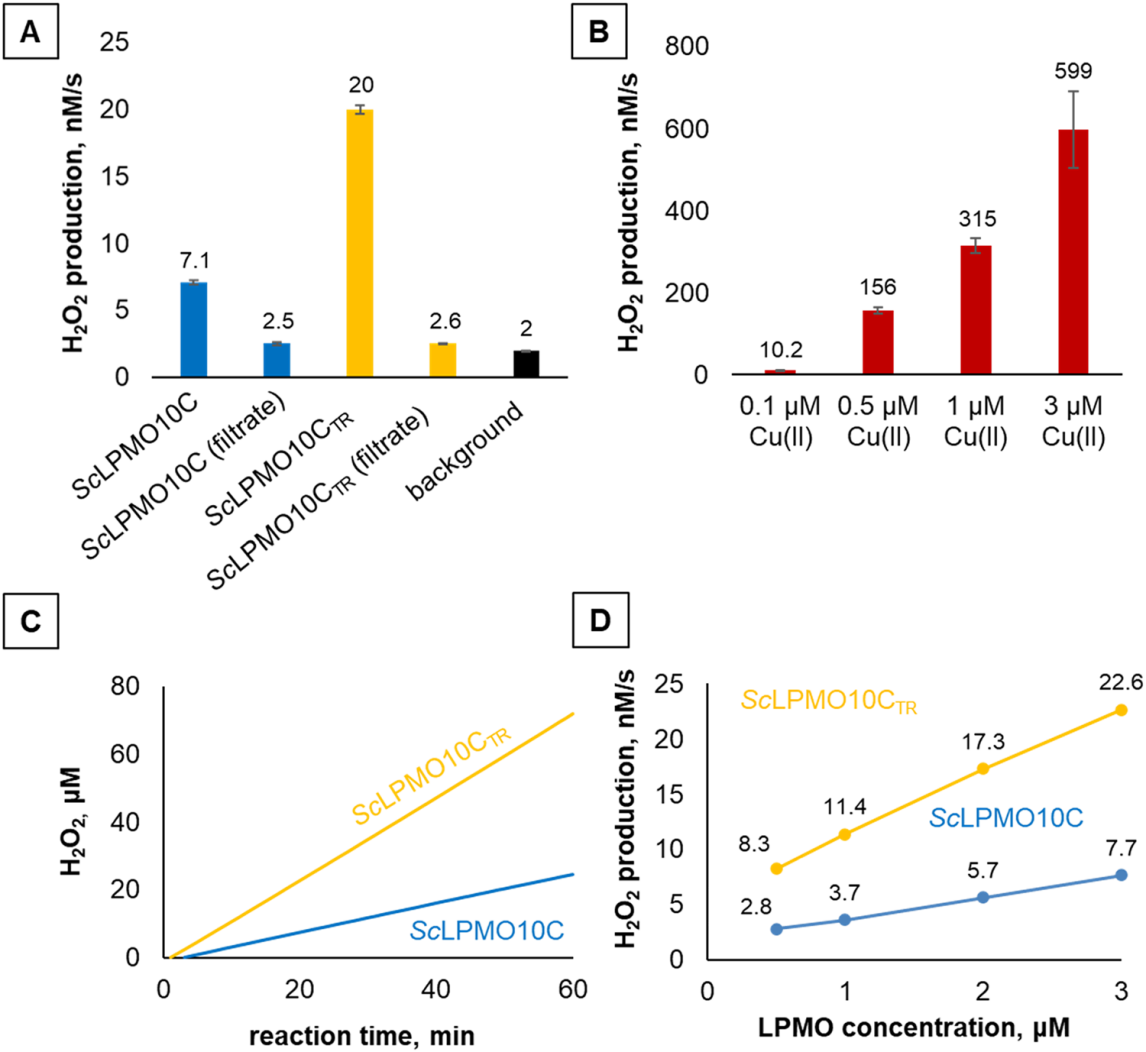
Apparent hydrogen peroxide production in reactions containing *Sc*LPMO10C and *Sc*LPMO10C_TR_ (panels **A**, **C**, **D**) or Cu(II)SO_4_ (panel **B**)_._ All experiments were carried out using 1 mM ascorbic acid in 50 mM sodium phosphate buffer, pH 6.0, at 30 °C. Reaction mixtures contained 5 U/ml HRP, 100 µM Amplex Red, 1% (v/v) DMSO and varying concentrations of LPMOs and Cu(II)SO_4_. The data shown in panels A and C are for LPMO concentrations of 3 μM. H_2_O_2_ production rates were derived from linear progress curves (such as shown in panel C; in this experiment the signal is monitored continuously). Free copper control experiments designated as “*Sc*LPMO10C filtrate” and “*Sc*LPMO10C_TR_ filtrate” in panel A were carried our using protein-free samples obtained from enzyme stock solutions by ultrafiltration. Such filtrates will contain the same amount of free copper ions as the LPMO preparations. The black bar in panel A denotes the background rate of hydrogen peroxide accumulation in the reaction containing reductant but lacking LPMO. Error bars indicate standard deviations between triplicates. The rates shown in panels A and D were obtained in independent experiments. Error bars in panel D are hidden behind the markers.

In a recent study by our group, we showed that some of the previously published activity data for *Sc*LPMO10C likely were affected by the presence of free copper in the reaction mixture, probably as a result of incomplete desalting of the enzyme preparations after copper-saturation^28^. It is well established that the combination of free Cu(II) ions and ascorbic acid will promote enzyme-independent H_2_O_2_ generation^21^, sometimes referred to as reductant autooxidation, which will boost LPMO activity and affect enzyme stability^28,36^. To ensure that the residual free copper levels in *Sc*LPMO10C and *Sc*LPMO10C_TR_ preparations used in the present study were minimal, protein-free samples were obtained from enzyme stock solutions by ultrafiltration through 3 kDa MWCO membranes. Analysis of these control samples with the HRP/Amplex Red assay, using 1 mM ascorbic acid as reductant, showed very low hydrogen peroxide production rates, compared to reactions with the LPMO (Fig. 2A), which demonstrates that the enzyme preparations contained negligible amounts of free copper.

Surprisingly, despite a comparable very low level of free copper in both enzyme preparations, as shown by the control reactions with the ultrafiltrates, the two enzyme variants (at 3 μM) showed a three-fold difference in the hydrogen peroxide generation rate (Fig. 2A). While it is reasonable to assume that these two enzymes generate different amounts of H_2_O_2_ in the presence of cellulose, due to variation in substrate binding affinity (as discussed in detail below), we expected to see equal performance of both LPMOs under the conditions of the HRP/Amplex Red assay (i.e., in the absence of any polysaccharide substrate).

Given the fact that even very low amounts of free copper will lead to significant H_2_O_2_ generation in this reaction set-up (Fig. 2B), one has to be extremely careful when interpreting the observed difference in H_2_O_2_ production between *Sc*LPMO10C and *Sc*LPMO10C_TR_. Looking for explanations, we first considered the possibility that the difference is caused by differences in the rate by which trace amounts of free copper (e.g., 0.1 μM copper, see Fig. 2B) are released from the active site of the LPMOs due to different rates of auto-catalytic damage. Release of copper from the LPMO active site under reducing conditions is conceivable since it is known that auto-catalytic oxidation predominately affects copper-binding histidine residues^16^. Importantly, as demonstrated by the control experiment depicted in Fig. 2B, the gradual accumulation of free copper ions in solution would cause the apparent rate of hydrogen peroxide generation to change over time. Since the H_2_O_2_-production curves obtained for both LPMO variants (Fig. 2C) are linear, it seems safe to conclude that free copper does not contribute significantly to the generation of H_2_O_2_ in this reaction set-up.

An additional control experiment with varying amounts of both LPMOs (0.5–3 μM) showed that the gap between the H_2_O_2_-generation rates is constant across all tested concentrations (Fig. 2D), which would not be the case if free copper is indeed released under the assay conditions at rates that differ between the two enzymes. Thus, it would seem that the observed difference in H_2_O_2_ production between *Sc*LPMO10C and *Sc*LPMO10C_TR_ relates to an intrinsic enzyme property, i.e., a difference in oxidase activity. A possible, although highly speculative, explanation is that the CBM weakly interacts with the substrate-binding surface of the catalytic domain, which could reduce oxidase activity by shielding the active site Cu(II) from the solvent and the reductant.

### Synergistic solubilization of cellulose by *Sc*LPMO10C and *Sc*LPMO10C_TR_

Our next goal was to compare the performance of *Sc*LPMO10C and *Sc*LPMO10C_TR_ during Avicel depolymerization. The hydrogen peroxide production gap between the full-length and truncated LPMO observed in the HRP/Amplex Red assay is likely to become more pronounced in the presence of cellulose. As alluded to above, existing data indicate that binding to polysaccharide substrates prevents LPMOs from producing hydrogen peroxide^27,28^. Furthermore, it is well established that the cellulose-binding module of *Sc*LPMO10C is capable of much stronger interaction with substrate than the catalytic domain of the enzyme, and is thus important for keeping the enzyme active site closer to its substrate^31,33^. Hence, it is sensible to expect enhanced in situ hydrogen peroxide generation by weak-binding *Sc*LPMO10C_TR_ compared to the stronger binding full-length LPMO during Avicel degradation.

**Figure 3:**
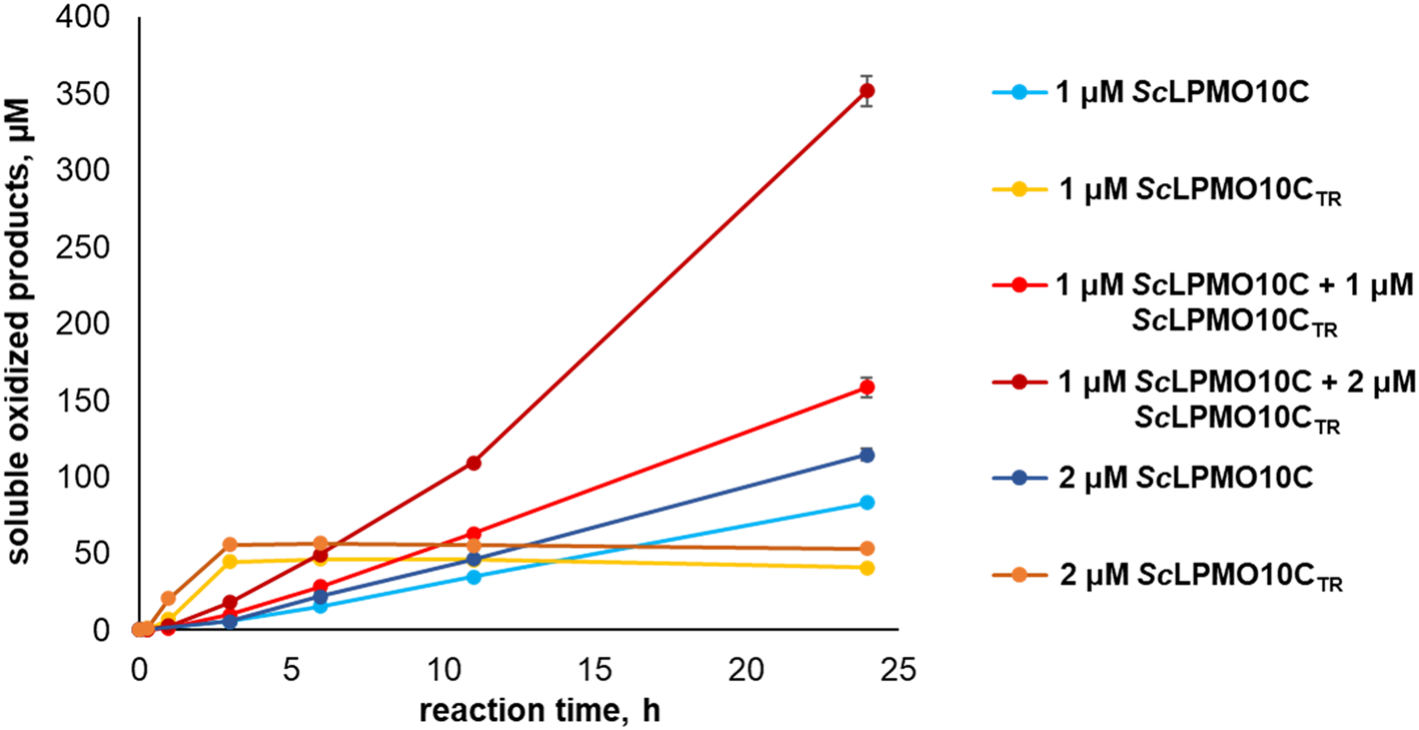
Cellulose oxidation by *Sc*LPMO10C and *Sc*LPMO10C_TR_. The graph shows soluble oxidized products released from 1% (w/v) Avicel by *Sc*LPMO10C, *Sc*LPMO10C_TR_ or mixtures of these LPMOs in the presence of 1 mM ascorbic acid. The reactions were carried out in 50 mM sodium phosphate buffer, pH 6.0, supplied with 1 mM ascorbic acid, at 30 °C. Error bars indicate standard deviations between triplicates.

Interestingly, if one accepts the premise that LPMO action is limited by access to H_2_O_2_, and if the above considerations about expected H_2_O_2_ production levels in the presence of substrate are true, it is conceivable that weak binding *Sc*LPMO10C_TR_ and stronger binding full-length *Sc*LPMO10C may act synergistically during Avicel degradation. The truncated LPMO will spent most of its time in solution, generating hydrogen peroxide to boost the oxidative activity of the wild-type enzyme that is anchored on a crystalline surface by its CBM. On the other hand, the truncated enzyme, which is prone to damaging off-pathway reactions^33^, may be protected by the full-length enzyme that removes H_2_O_2_ from the solution in productive reactions. To test this hypothesis, we carried out Avicel degradation experiments with *Sc*LPMO10C, *Sc*LPMO10C_TR_ or a combination of both LPMOs in the presence of ascorbic acid (Fig. 3).

Cellulose depolymerization by 1 or 2 μM *Sc*LPMO10C resulted in a slow (0.05 min^-1^) but steady release of oxidized products over 24 h. Such low catalytic rates are common among bacterial LPMOs operating in the absence of exogenously added hydrogen peroxide^1,18,28^. In contrast to the full-length enzyme, the reactions with 1 or 2 μM *Sc*LPMO10C_TR_ were much (approximately fourfold) faster. In these reactions, product release diminished early during the course of the experiment. This is indicative of LPMO auto-catalytic inactivation. It is well known that LPMO instability is promoted by reduced binding to the substrate^37^ and high levels of H_2_O_2_^16^, and both apply to *Sc*LPMO10C_TR_ relative to the full-length enzyme.

Strikingly, reactions with 1 μM *Sc*LPMO10C + 1 μM *Sc*LPMO10C_TR_ or 1 μM *Sc*LPMO10C + 2 μM *Sc*LPMO10C_TR_ did not show signs of enzyme inactivation and generated higher product levels compared to reactions with the individual LPMOs (Fig. 3). The amount of oxidized products released after 24 h was 1.3-fold (1 μM *Sc*LPMO10C + 1 μM *Sc*LPMO10C_TR_) and 2.6-fold (1 μM *Sc*LPMO10C + 2 μM *Sc*LPMO10C_TR_) higher compared to the sum of the products generated by these enzymes in individual reactions, indicative of a synergistic action. The reaction with 1 μM *Sc*LPMO10C + 2 μM *Sc*LPMO10C_TR_ is remarkable, showing a much higher rate than *Sc*LPMO10C alone, no signs of enzyme inactivation and high product levels. Considering the observed (Fig. 2) and expected H_2_O_2_ production levels, the increased rate of the reaction may be explained by increased levels of available H_2_O_2_, which is produced by the truncated enzyme. At the same time, the truncated enzyme is protected from damaging off-pathway reactions with H_2_O_2_ because the oxidant is used in productive reactions by the full-length enzyme. There are indications, from modelling^38^ and experiments^39^, that binding of H_2_O_2_ by an LPMO is promoted by the presence of substrate. Thus, it is plausible that the full-length enzyme, with high substrate affinity, outcompetes the truncated enzyme, with low substrate affinity, for binding (and reacting with) H_2_O_2_.

Paradoxically, the initial rate of the most efficient reaction (1 μM *Sc*LPMO10C + 2 μM *Sc*LPMO10C_TR_) was still considerably lower than the initial rate of the reaction with 1 μM or 2 μM *Sc*LPMO10C_TR_ alone (Fig. 3, first three hours). Looking for possible explanations, it is important to take into account the expected instability of *Sc*LPMO10C_TR_, which may lead to gradual release of copper into solution, which will boost the activity of still intact LPMOs because free copper promotes LPMO-independent H_2_O_2_ generation in the presence of ascorbic acid (Fig. 2B). In other words, the decreased initial rate of product release observed upon adding *Sc*LPMO10C to a reaction with *Sc*LPMO10C_TR_, may be a direct consequence of the above-mentioned stabilization of the truncated enzyme. This complicated scenario is further explained and experimentally verified below.

### Reductant consumption in reactions with *Sc*LPMO10C and *Sc*LPMO10C_TR_

If copper is indeed released from the active site of unstable LPMOs then this effect should be carefully accounted for while interpreting the data shown on Fig. 3. Thus, our next goal was to evaluate whether spectroscopic quantification of ascorbic acid in LPMO reactions can be used as a tool to detect leakage of copper ions from the oxidatively damaged histidine brace. At standard aerobic conditions, the depletion of reductant is driven by LPMO-dependent H_2_O_2_ production (oxidase activity), LPMO-dependent substrate oxidation (peroxygenase activity), reactions between hydrogen peroxide and ascorbic acid^25^ and enzyme-independent H_2_O_2_ generation (i.e., oxidation of the reductant by O_2_). For some reductants, including ascorbic acid, enzyme-independent H_2_O_2_ generation is strongly promoted by free copper^28^. Indeed, under the reaction conditions used here, major effects of free copper will occur at concentrations (e.g., 0.1 μM; (Fig. 2B) that are well below the LPMO concentrations (1–3 μM) used in this study. Thus, copper release caused by oxidative damage to the active site of only a fraction of the LPMOs could strongly affect the activity (and exposure to H_2_O_2_) of the remaining intact enzymes.

**Figure 4:**
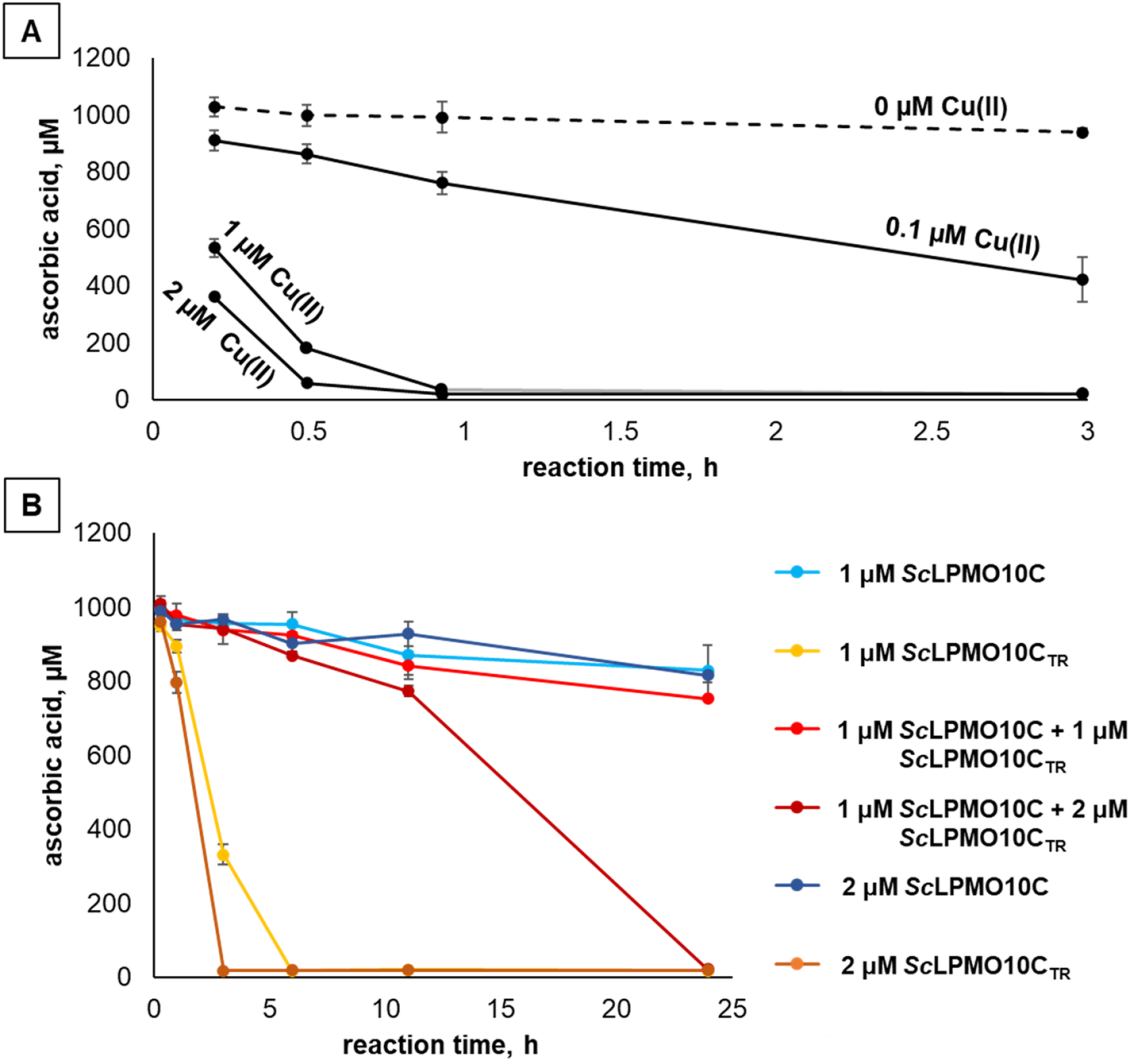
Ascorbic acid consumption in reactions containing Cu(II)SO_4_ (panel **A**) or LPMOs (panel **B**). The reactions were carried out at 30 °C in 50 mM sodium phosphate buffer, pH 6.0, supplied with 1% (w/v) Avicel, and were initiated by the addition of 1 mM ascorbic acid at t = 0. The reductant concentration was determined by UV spectroscopy immediately after removing the insoluble LPMO substrate by filtration. Note that the reductant depletion curves in panel B were obtained using the same samples as shown in Fig. 3. Error bars indicate standard deviations between triplicates.

Figure 4A shows the impact of free copper on reductant depletion under the conditions used for the reactions shown in Figs. 2 and 3. In accordance with Fig. 2B, showing increased H_2_O_2_ production upon addition of 0.1 μM Cu(II), Fig. 4A shows that the presence of 0.1 μM Cu(II) drastically speeds up the turnover of ascorbic acid. Thus, there is no doubt that under the conditions used here, which are often applied in the field, release of copper from damaged LPMOs will lead to increased H_2_O_2_ production and, thus, fueling of the remaining intact LPMOs.

Quantification of ascorbic acid in reactions with substrate (i.e., the reactions displayed in Fig. 3) revealed slow and steady reductant depletion in the reactions with 1 μM *Sc*LPMO10C, 2 μM *Sc*LPMO10C or 1 μM *Sc*LPMO10C + *Sc*LPMO10C_TR_ (Fig. 4B), which coincides with the close to linear product formation progress curves of Fig. 3. In stark contrast, the reactions with 1 or 2 μM *Sc*LPMO10C_TR_ showed rapid and complete ascorbic acid consumption within first 6 or 3 h of the experiment, respectively (Fig. 4B). Notably, this high consumption is nowhere near to being proportional to the low amounts of oxidized LPMO products released in these reactions (Fig. 3), showing that reducing power is used in another process, for example reduction of O_2_ to H_2_O_2_. The reductant depletion rates observed with 1 or 2 μM *Sc*LPMO10C_TR_ are comparable to the results of the control experiments with free Cu(II) (Fig. 4A) and strongly suggest that copper indeed leaves the LPMO active site, which will increase H_2_O_2_ production in the presence of ascorbic acid.

Thus, we propose, that the high initial rate and fast enzyme inactivation observed in reactions with 1 or 2 μM *Sc*LPMO10C_TR_ (Fig. 3) are the result of a self-reinforcing process: The LPMO binds weakly to the substrate and produces lots of H_2_O_2_; this leads to higher LPMO activity and higher LPMO inactivation, where the latter leads to the release of free copper, which, by promoting H_2_O_2_ production, further increases LPMO activity and LPMO inactivation. The result is a high initial rate of reaction and fast cessation of the reaction, in accordance with the progress curves shown in Fig. 3. This self-reinforcing process will be hindered upon addition of the full-length LPMO that uses the H_2_O_2_ in productive LPMO reactions, which explains the paradox drawn up in the previous section.

It may seem contradictory that, even in the presence of substrate, *Sc*LPMO10C_TR_ shows signs of inactivation (reflected in excessive ascorbic acid consumption) within 1 h of the reaction (Fig. 4B), whereas this enzyme gave a linear H_2_O_2_ production curve in the experiment depicted in Fig. 2C. Importantly, in this latter experiment, generated H_2_O_2_ is rapidly consumed by the HRP present in the reaction, which will drastically reduce enzyme inactivation and the consequent release of copper that promotes reductant consumption.

Importantly, while cessation of product formation in LPMO reactions is usually ascribed to enzyme inactivation, the data in Fig. 4 illustrate, that, due to the self-reinforcing nature of the LPMO inactivation process, fast depletion of ascorbic acid may occur, which will also stop the LPMO reaction. In the reactions shown here, both factors may have played a role. However, the progress curves for the reaction with 1 μM *Sc*LPMO10C_TR_ indicate that product formation stopped at or before 3 h (Fig. 3), while the reductant had not yet been completely depleted (Fig. 4B). Hence, in this experiment, oxidative inactivation of the LPMO was the dominating process leading to cessation of the reaction.

**Figure 5:**
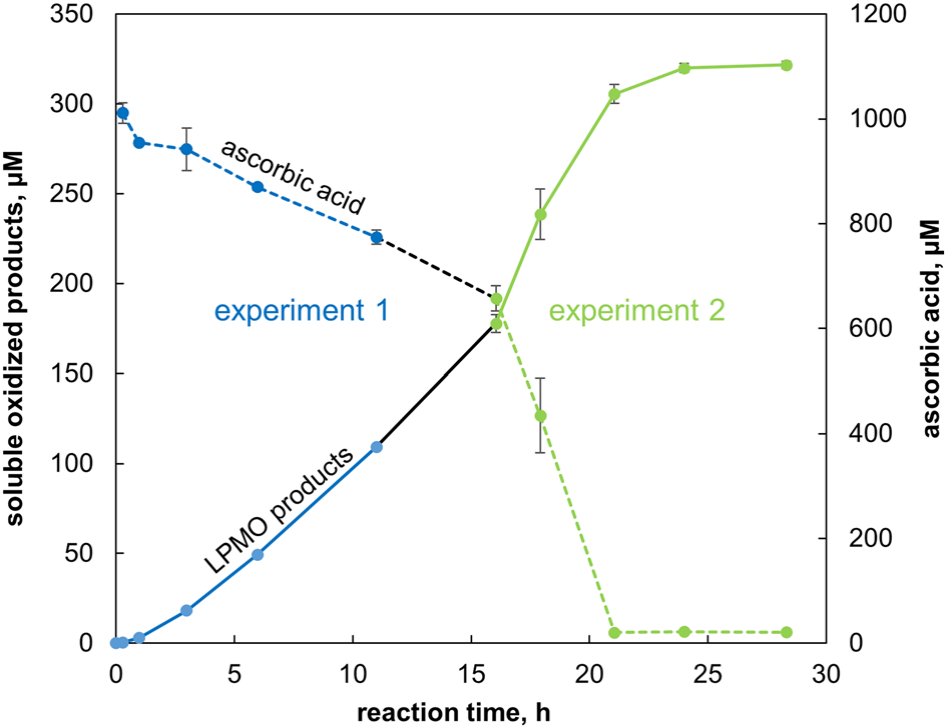
Release of oxidized products and reductant depletion in the reaction with 1 µM*Sc*LPMO10C + 2 µM *Sc*LPMO10C_TR_. The reactions were carried out at 30 °C in 50 mM sodium phosphate buffer, pH 6.0, supplied with 1% (w/v) Avicel and were initiated by adding 1 mM ascorbic acid. The data shown in blue and green color represent two independent experiments. The blue progress curves (“experiment 1”) are the same as shown in Figs. 3 and 4B. In the second identical experiment (green) only later time points were monitored. The black lines connect the blue curves with the green curves. The reductant concentration was determined by UV spectroscopy immediately after removing the insoluble LPMO substrate by filtration. Error bars indicate standard deviations for triplicates.

More insight into this matter may be derived from the ascorbic acid depletion curve for the reaction with 1 μM *Sc*LPMO10C + 2 μM *Sc*LPMO10C_TR_, which displayed an apparent biphasic behavior (Fig. 4B). A slow (i.e., enzyme-dependent) reductant consumption phase was followed by a phase with a faster decrease in the ascorbic acid concentration. Expansion of the progress curves shown in Figs. 3 and 4 with additional data points confirmed the biphasic nature of the process. Figure 5 shows that the phase with steady ascorbic acid consumption was followed by a phase of rapid reductant consumption and cessation of product formation that reflects a collapse of the reaction system, similar to what was seen for the reactions with *Sc*LPMO10C_TR_ only, where this collapse happened earlier during the reaction due to the absence of the H_2_O_2_-consuming full-length enzyme (Fig. 4B).

The only plausible explanation for the collapse observed in this biphasic reaction is the release of copper by damaged LPMOs. Under turnover conditions gradual LPMO inactivation and copper-release from damaged LPMOs are to be expected due to off-pathway reactions, even though, in well controlled reaction systems, off-pathway reactions may be infrequent and not necessarily always destructive for the LPMO^40^. It is conceivable that at about 15 h in the biphasic reaction accumulated copper reached levels that triggered the self-reinforcing enzyme inactivation process described above. This process could initially speed up the reaction rate (as is visible in Fig. 5, showing a vaguely sigmoidal curve for product formation) but will lead to system collapse and rapid ascorbic acid depletion. Product formation (Fig. 3) and ascorbic acid depletion (Fig. 4B) remained constant for the full 24 h of the reaction containing 50% less of the truncated LPMO (1 μM *Sc*LPMO10C + 1 μM *Sc*LPMO10C_TR_ reaction). So, in this reaction, where one would expect less H_2_O_2_ production and less enzyme inactivation, the turning point leading to rapid ascorbic acid depletion and biphasic behavior was never reached because there was less release of free copper into solution.

### Stability of *Sc*LPMO10C and *Sc*LPMO10C_TR_ in the absence of substrate

**Figure 6:**
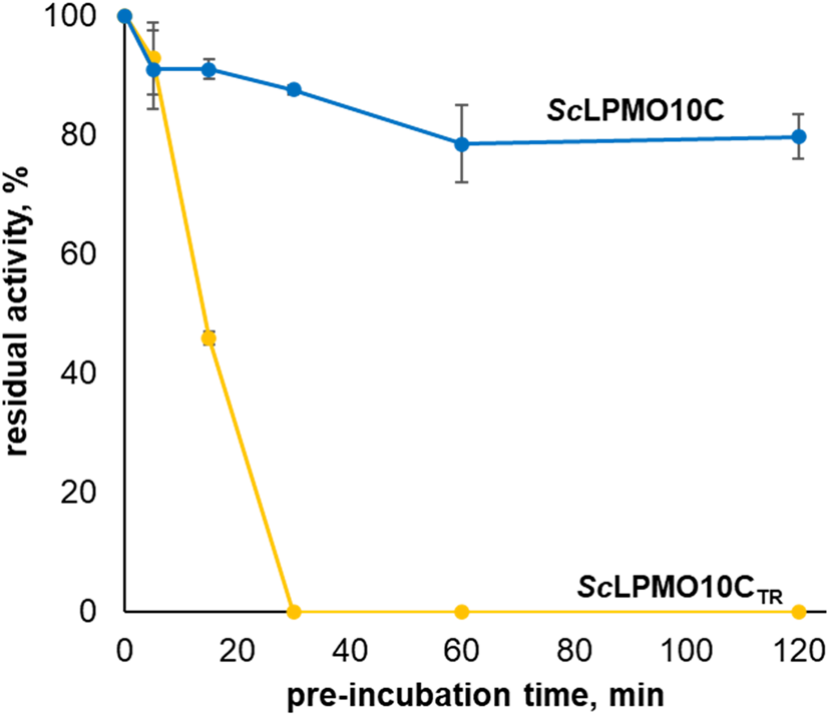
Oxidative stability of *Sc*LPMO10C and *Sc*LPMO10C_TR_. The figure shows the residual activity of 1 µM *Sc*LPMO10C and 1 µM *Sc*LPMO10C_TR_ in a reaction with 1% (w/v) Avicel after various periods of pre-incubation with 1 mM ascorbic acid in the absence of substrate (i.e., under conditions that promote auto-catalytic inactivation). The cellulose degradation reaction was carried out for 24 h at 30 °C in 50 mM sodium phosphate buffer, pH 6.0, supplied with fresh 1 mM ascorbic acid. Error bars indicate standard deviations between triplicates.

In line with previously published data, the above is based on the assumption that inactivation of an LPMO in the absence of substrate will relate to the oxidase activity of that LPMO (i.e., H_2_O_2_ production). To verify this assumption, we assessed the stability of the full-length and truncated enzymes under reducing conditions in the absence of substrate. 2 μM *Sc*LPMO10C or 2 μM *Sc*LPMO10C_TR_ were pre-incubated with 1 mM ascorbic acid for 2 h to promote inactivation. Aliquots were taken at various time points and then supplied with Avicel and another (the same) dose of ascorbic acid to assess the residual activity. This approach is not without limitations because free copper ions that have left the histidine brace as a result of enzyme inactivation will affect the subsequent LPMO reaction with Avicel where free copper will affect both activity and stability. Nevertheless, the results, depicted in Fig. 6, show a clear difference: the full-length LPMO retained most of its catalytic potential during the pre-incubation, whereas the truncated enzyme was rapidly inactivated. This difference is expected in light of the difference in H_2_O_2_ production by the two enzymes (Fig. 2A). Taken together, the results shown in Figs. 2 and 6 suggest that truncation of the CBM significantly impairs the oxidative stability of *Sc*LPMO10C in the absence of substrate, which is remarkable.

### Concluding remarks

This study provides insight into the interplay between LPMOs with different H_2_O_2_ generation and consumption capabilities. Being a much better hydrogen peroxide producer, *Sc*LPMO10C_TR_ is able to enhance cellulose oxidation by *Sc*LPMO10C. At the same time, *Sc*LPMO10C protects *Sc*LPMO10C_TR_ from fast auto-catalytic inactivation by removing excess H_2_O_2_. The synergistic action of the two LPMOs allowed for steady cellulose depolymerization over 24 h, with an efficiency that clearly surpassed the efficiencies of the individual enzymes.

While these results shed light on the complexity of LPMO reactions, especially when using “copper-sensitive” reductants, it remains to be seen whether the synergy observed here also occurs in Nature. LPMOs are abundant and many organisms contain multiple LPMOs, some with and some without CBMs. These CBM-free LPMOs could help fueling other LPMOs with H_2_O_2_ as shown above, and could also fuel other H_2_O_2_-dependent enzymes such as lignin peroxidases^41^. On the other hand, in contrast to the catalytic domain of CBM-containing *Sc*LPMO10C, these natural single domain LPMOs may very well have evolved to bind their substrates strongly, as observed for, e.g., the archetypal single domain LPMO *Sm*LPMO10A (or CBP21;^39^), and may thus be less efficient H_2_O_2_-producers.

While we expected relatively fast H_2_O_2_ generation by *Sc*LPMO10C_TR_ in reactions with cellulose because of low binding, the truncated LPMO was found to produce more hydrogen peroxide than the full-length enzyme even in the absence of substrate. As we show and discuss above, this surprising result is not driven by free copper and thus seems related to hitherto unknown effects of the CBM on LPMO oxidase activity. It is possible that the higher H_2_O_2_ production rate of *Sc*LPMO10C_TR_ in the absence of substrate is an artifact of LPMO truncation, which, in principle could lead to minor changes in the enzyme structure that could make the reduced copper more prone to oxidation by O_2_. However, this seems unlikely, given the fact that the truncation point is > 35 Å away from the active site and that the X-ray structure of *Sc*LPMO10C_TR_ shows no anomalies^35^ (PDB: 4OY7). Another possible explanation is that the catalytic and cellulose-binding domains of *Sc*LPMO10C interact in an inter-molecular or intra-molecular fashion. It is tempting, although highly speculative, to propose that the CBM may protect the LPMO from auto-catalytic damage in the absence of substrate by shielding the active site from the reductant (preventing reduction), or from reacting with O_2_, thus keeping the fraction of H_2_O_2_-producing enzyme molecules low. Further studies are needed to explore this scenario.

In addition to providing insights into the possible impact of CBMs on LPMO functionality and generating more insight into the crucial role of H_2_O_2_ in LPMO catalysis, the present data shed light on the complexity of interpreting ascorbic acid-driven LPMO reactions. Ascorbic acid was, somewhat fortuitously, used in research leading to the discovery of LPMO activity^1^ and has been used by many researchers since. This “copper-sensitive” reductant may engage in multiple reactions that are difficult to control and assess. One of these reactions, the scavenging of excess H_2_O_2_ (i.e., further reduction to water) was not considered in the above because there is ample data showing that this reaction is slow relative to the other possible reactions^17,40^.

Progress curves for LPMO reactions with ascorbic acid often show apparent enzyme inactivation and the present study reveals, to the best of our knowledge for the first time, a plausible underlying mechanism. We show that release of copper ions as a consequence of (well known;^16,37^) oxidative damage to the LPMO active site triggers a self-reinforcing chain reaction leading to increased enzyme-independent H_2_O_2_ generation, rapid enzyme inactivation and reductant depletion. Similar processes may occur in reactions with other reductants whose ability to reduce O_2_ is copper-dependent, such as L-cysteine^23^ and reduced glutathione^42^. While fully controlling and understanding all these processes remains challenging, the present study underpins that optimization of LPMO activity and stability requires control of H_2_O_2_ levels and that, in many commonly used experimental settings, these levels are affected not only by LPMO oxidase activity but also by LPMO inactivation. Since oxidase activity varies between LPMOs^17,43^ optimal conditions for LPMO reactions, and the relative impact of free copper, will vary.

## Methods

### Materials

Chemicals were obtained from Sigma-Aldrich (St. Louis, MO, USA) unless specified otherwise. The model microcrystalline cellulose substrate used in the study was Avicel PH-101 with particle size of approximately 50 µm. 100 mM ascorbic acid stock solutions were prepared in metal-free TraceSELECT water (Honeywell, Charlotte, NC, USA) and then filter-sterilized using 0.22 µm syringe filters. Amplex Red was purchased from Thermo Fisher Scientific (Waltham, MA, USA) and dissolved in DMSO at 10 mM final concentration. The reductant and Amplex Red solutions were aliquoted, stored at −20 °C in light-protected tubes and used only once after thawing. Horseradish peroxidase type II (HRP; Sigma-Aldrich, St. Louis, MO, USA) was kept at 100 U/ml concentration in 50 mM sodium phosphate buffer, pH 6.0 at 4 °C. Tryptone and yeast extract were obtained from Thermo Fisher Scientific.

### Protein production and purification

Previously generated *Sc*LPMO10C and *Sc*LPMO10C_TR_ expressing *E. coli* BL21 DE3 strains^35^ were used to inoculate Terrific Broth (TB) medium supplied with 100 µg/ml ampicillin. The cultures (2 × 500 ml for each LPMO-expressing strain) were incubated for 24 h at 30 °C in a LEX-24 Bioreactor (Harbinger Biotechnology & Engineering, Markham, Canada) using compressed air for aeration. The cells were harvested by centrifugation (6000 × g for 10 min at 4 °C) using a Beckman Coulter centrifuge (Brea, CA, USA). Periplasmic extracts were produced by applying osmotic shock as described previously^44^ and were filter-sterilized through 0.22-µm syringe filters prior to further purification. These extracts were then subjected to ion-exchange chromatography using HiTrap Q FF (Q Sepharose) 5-ml columns (GE Healthcare, Chicago, USA). Proteins were eluted at 2.5 ml/min flow rate with a linear gradient of NaCl (0–500 mM, 250 ml) in the starting buffer (50 mM Tris–HCl, pH 7.5). LPMO-containing fractions were pooled, concentrated to approximately 1 ml volume (Vivaspin ultrafiltration tubes, 10 kDa MWCO, Sartorius, Göttingen, Germany) and loaded onto a ProteoSEC Dynamic 16/60 3–70 HR preparative size-exclusion column (Protein Ark, Sheffield, UK) operating at 1 ml/min flow rate and equilibrated with 50 mM Tris-HCl, pH 7.5, containing 200 mM NaCl. Purity of resulting *Sc*LPMO10C and *Sc*LPMO10C_TR_ preparations was confirmed by SDS-PAGE. The LPMO concertation was determined using UV spectroscopy at 280 nm. The theoretical extinction coefficients were predicted using the Expasy ProtParam tool^45^.

### Copper saturation

Purified LPMO solutions were concentrated to 0.5 ml volume using Vivaspin ultrafiltration tubes (10 kDa MWCO, Sartorius, Göttingen, Germany) and incubated with two-fold molar surplus of Cu(II)SO_4_ at room temperature for 30 min. The excess (unbound) copper was removed by desalting with a PD MidiTrap G-25 gravity flow column (GE Healthcare, Chicago, USA), equilibrated with 50 mM sodium phosphate buffer, pH 6.0. To ensure effective free copper removal, the *Sc*LPMO10C and *Sc*LPMO10C_TR_ samples were then subjected to multiple consecutive rounds of dilution and concertation using 50 mM sodium phosphate buffer, pH 6.0 and Vivaspin ultrafiltration tubes. The resulting total dilution factors for solutions of copper-saturated LPMOs amounted to at least 160 000.

### Hydrogen peroxide detection

In situ hydrogen peroxide generation in LPMO reactions was assessed using a modified HRP/Amplex Red assay protocol^18^, originally proposed by Kittl et al.^24^. In brief, 90 µl of LPMO solution in 50 mM sodium phosphate buffer, pH 6.0, supplied with HRP and Amplex Red, was pre-incubated for 5 min at 30 °C in a 96-well microtiter plate. Next, 10 µl of 10 mM ascorbic acid was added to start the reaction, followed by 10 s of mixing at 600 RPM inside a Varioscan LUX plate reader (Thermo Fisher Scientific, Waltham, MA, USA). Hydrogen peroxide was detected by monitoring the formation of resorufin at 563 nm. The final concentrations of LPMOs, HRP, Amplex Red and ascorbic acid were 0.5–3 µM, 5 U/ml, 100 µM and 1 mM, respectively. H_2_O_2_ standard solutions were prepared in 50 mM sodium phosphate buffer, pH 6.0, and supplied with 1 mM ascorbic acid prior to the addition of HRP and Amplex Red. The background non-enzymatic H_2_O_2_ production was assessed in a control experiment with 1 mM ascorbic acid and no LPMO.

To ensure that the observed LPMO-dependent hydrogen peroxide generation is not biased by residual free copper ions in enzyme preparations, protein-free samples were obtained from the LPMO stock solutions using 3 kDa MWCO 1.5 ml ultrafiltration tubes (VWR International, Radnor, PA, USA) and were used to set up control experiments by substituting LPMOs with the same amount of filtrates, as previously described^28^.

### LPMO reactions with microcrystalline cellulose

LPMO reactions with insoluble substrate (1% w/v Avicel) were carried out in 50 mM sodium-phosphate buffer, pH 6.0 using an Eppendorf thermomixer (Eppendorf, Hamburg, Germany) set to 30 °C and 950 RPM. The reactions were started by supplying 1 mM ascorbic acid (final concentration).

Aliquots were taken at various time points, and the reactions were quenched by removing the insoluble substrate using a 96-well filter plate (Millipore, Burlington, MA, USA). The concentration of ascorbic acid in filtered solutions was determined by UV spectrometry at 255 nm wavelength. Standard solutions of the reductant were prepared in metal-free TraceSELECT water. The samples were then treated with 2 µM of recombinant *Thermobifida fusca* GH6 endoglucanase (*Tf*Cel6A; produced in-house)^46^ at 37 °C overnight to convert soluble C1-oxidized oligosaccharides to a simple mixture of oxidized dimers and trimers (i.e. GlcGlc1A and Glc_2_Glc1A).

### LPMO oxidative stability assay

The oxidative stability of *Sc*LPMO10C and *Sc*LPMO10C_TR_ was assessed by pre-incubating 2 μM enzyme in 50 mM sodium phosphate buffer, pH 6.0, containing 1 mM ascorbic acid, for two hours at 30 °C. Aliquots were taken at various time points and supplied with 1% (w/v) Avicel and another (the same) dose of ascorbic acid. LPMO reactions with Avicel were carried out for 24 h and then stopped by filtering out the insoluble substrate. These samples were treated with *Tf*Cel6A as described above and used to quantify the residual LPMO activity. The final concentration of both LPMOs in the reactions with Avicel was 1 µM.

### Quantification of LPMO products

C1-oxidized soluble LPMO products were detected and quantified by high-performance anion-exchange chromatography with pulsed amperometric detection (HPAEC-PAD) using a Dionex ICS6000 system (Thermo Scientific, San Jose, CA, USA) equipped with a CarboPac PA200 analytical column, as recently described^47^. In brief, a concave gradient from 1 to 100 mM potassium methanesulfonate (KMSA) was applied over 14 min with 63 μl/min flow rate to separate native and C1-oxidized oligosaccharides, followed by a 3 min washing step (100 mM KMSA) and a 9 min column reconditioning step (1 mM KMSA). LPMO products were quantified using standard mixtures of C1-oxidized cellobiose and cellotriose, which were produced in-house using cellobiose dehydrogenase^48^, according to a previously published protocol^49^.

## Supplementary Information

Supplementary Information.

## Data Availability

The data that support the findings of this study are available in Figs. 1–6.
