# Supplementary material for: Enhanced in situ H2O2 production explains synergy between an LPMO with a cellulose-binding domain and a single-domain LPMO
Source: Sci Rep. 2022 Apr 12;12:6129. doi: 10.1038/s41598-022-10096-0 (PMC9005612; doi:10.1038/s41598-022-10096-0)
Supplement: Supplementary file 1 — Supplementary Information. [file 41598_2022_10096_MOESM1_ESM.pdf]

# Enhanced in situ $H_2O_2$ production explains synergy between an LPMO with a cellulose-binding domain and a single-domain LPMO

Anton A. Stepnov[1], Vincent G. H. Eijsink[1*], Zarah Forsberg[1*]

[1] - Faculty of Chemistry, Biotechnology and Food Science, NMBU - Norwegian University of Life Sciences, 1432 Ås, Norway

[*] corresponding authors; vincent.eijsink@nmbu.no, zarah.forsberg@nmbu.no

## Supplementary information

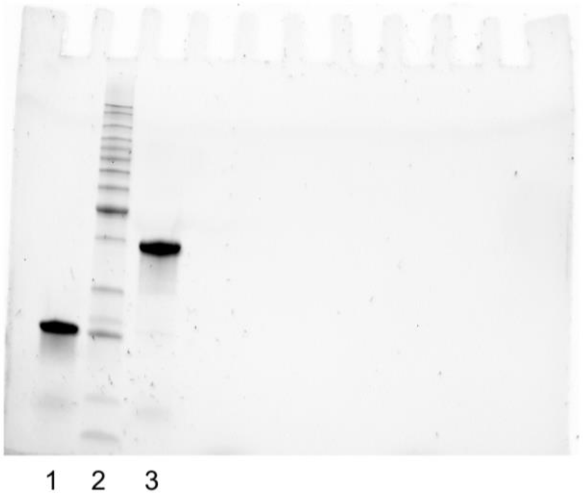


**Figure S1. SDS-PAGE of *Sc*LPMO10C$_{TR}$ and *Sc*LPMO10C after purification.** The figure shows the uncropped and unprocessed gel image used to create panel B in Fig. 1. Lane 1, 3 µg of *Sc*LPMO10C$_{TR}$; lane 2, commercial molecular weight marker (BenchMark™ Protein Ladder, Thermo Fisher Scientific, Waltham, MA, USA); lane 3, 3 µg of *Sc*LPMO10C.
